# Laminar Distribution of the Pathological Changes in Sporadic and Variant Creutzfeldt-Jakob Disease

**DOI:** 10.4061/2011/236346

**Published:** 2010-12-16

**Authors:** R. A. Armstrong

**Affiliations:** Department of Vision Sciences, Aston University, Birmingham B4 7ET, UK

## Abstract

The laminar distributions of the pathological changes in the cerebral cortex were compared in the prion diseases sporadic Creutzfeldt-Jakob disease (sCJD) and variant CJD (vCJD). First, in some cortical regions, the vacuolation (“spongiform change”) was more generally distributed across the cortex in sCJD. Second, there was greater neuronal loss in the upper cortex in vCJD and in the lower cortex in sCJD. Third, the “diffuse” and “florid” prion protein (PrP^sc^) deposits were more frequently distributed in the upper cortex in vCJD and the “synaptic” deposits in the lower cortex in sCJD. Fourth, there was a significant gliosis mainly affecting the lower cortex of both disorders. The data suggest that the pattern of cortical degeneration is different in sCJD and vCJD which may reflect differences in aetiology and the subsequent spread of prion pathology within the brain.

## 1. Introduction

Four subtypes of the prion disease Creutzfeldt-Jakob disease (CJD) have been described to date including familial CJD (fCJD), linked to germline mutations of the prion protein (*PrP*) gene [1], and iatrogenic CJD (iCJD), a transmissible form of the disease in which the disease form of prion protein (PrP^sc^) is acquired from growth hormone, dura mater grafts, or corneal transplantation [2]. By contrast, sporadic CJD (sCJD), the commonest form of the disease, may arise as a result of random mutation or posttranslational modification of the *PrP* gene [3, 4]. In addition, a new type of CJD, namely, variant CJD (vCJD), has been described in the United Kingdom [5]. Variant CJD differs significantly from previously described subtypes of the disease in having a younger age of onset (median age 23 years, range: 18–53 years), a prolonged duration of disease (12 to 24 months), a psychiatric presentation of the disease, and absence of typical EEG features [5]. Variant CJD has been linked to the consumption of meat originating from cattle with bovine spongiform encephalopathy (BSE) [5].

Neuropathologically, CJD is characterized by the presence in the brain of vacuolation “spongiform change”, neuronal loss, a reactive astrocytosis, and the deposition of PrP^sc^ in the form of discrete deposits or plaques [6, 7]. There are different morphological types of PrP^sc^ deposit in CJD. Hence, “florid” deposits are composed of a central “core” surrounded by a rim of small vacuoles and are especially characteristic of vCJD. Broad bundles of amyloid are present within the core, and the deposits resemble more closely the “classic” deposits typical of Alzheimer's disease (AD) [8] rather than the “kuru”-type deposits characteristic of prion disease [9]. In addition, there are more “diffuse-type” deposits in vCJD (also known as “fine feathery diffuse deposits” or “fine diffuse plaques”) [10] which are less aggregated, more weakly stained, and lack a distinct core [11]. By contrast, in the commonest type of sCJD, PrP^sc^ occurs in the form of “synaptic-type” deposits, and there are relatively few florid deposits or kuru plaques [12, 13]. 

Differences in pathology between subtypes of CJD could reflect variations in aetiology and the subsequent spread of prion pathology within the brain. Various hypotheses have been proposed to explain how PrP^sc^ spreads into the brain in CJD, for example, direct neural transmission from the site of infection, replication of PrP^sc^ in the spleen followed by neural entry through the spinal cord [14], infection of gut-associated lymphoid tissue with subsequent spread to the dorsal motor nucleus of the vagus nerve [15, 16], and spread via the circulatory system [17]. In neurodegenerative disorders, such as AD, dementia with Lewy bodies (DLB), and Pick's disease (PiD), the density of the pathological changes within the cerebral cortex often varies significantly across the different cortical laminae [18–20]. Neocortical regions are characterized as having six laminae (I to VI), each of which has a specific pattern of connections. Moreover, the laminar distribution of a pathological change may reflect the degeneration of specific anatomical pathways which have their cells of origin or axon terminals within particular cortical laminae [21]. Hence, the present study compared the laminar distributions of the pathological changes in cases of sCJD and vCJD to determine whether the pattern of cortical degeneration was different in the two prion disorders.

## 2. Materials and Methods

### 2.1. Cases

Eleven cases each of sCJD and vCJD (details in Table 1) were studied at the Brain Bank, Department of Neuropathology, Institute of Psychiatry (IOP), King's College London, UK. Informed consent was obtained for the removal of all brain material according to the 1999 Declaration of Helsinki (as modified Edinburgh 2000). Brain material of sCJD was obtained from the IOP and of vCJD from the National CJD Surveillance Centre, Western General Hospital, Edinburgh, UK. All cases fulfilled the neuropathological diagnostic criteria for CJD [22]. None of the cases had any of the known mutations of the *PrP* gene or family history of prion disease, and there was no evidence of the known types of iatrogenic aetiology. In the vCJD cases, the pattern of PrP^sc^ deposition typical of these cases was observed with florid-type PrP^sc^ deposits in the cerebral cortex, cerebellum, basal ganglia, thalamus, and brain stem [23, 24]. In addition, sCJD is classified according to heterogeneity at the polymorphic codon 129 of the *PrP* gene and the presence of Type 1 or Type 2 isoforms of PrP^sc^ [13]. The present cases conformed to the commonest subtype of sCJD, that is, homozygous for methionine at codon 129 and with Type 1 PrP^sc^ (M/M1). All vCJD cases studied were methionine/methionine (M/M) homozygotes at codon 129. In addition, the PrP^sc^ characteristic of vCJD had a uniform glycotype (PrP^sc^ Type 4) [23].

### 2.2. Histological Methods

Blocks of the frontal cortex (B8) at the level of the genu of the corpus callosum, parietal cortex (B7) at the level of the splenium of the corpus callosum, occipital cortex including the calcarine sulcus, inferior temporal gyrus (B22), and parahippocampal gyrus (B28) were taken from each case. Tissue was fixed in 10% phosphate-buffered formal saline and embedded in paraffin wax. Sequential, coronal 7 *μ*m sections were stained with haematoxylin and eosin (H/E), cresyl violet, or immunostained against PrP using the monoclonal antibody 12F10 (dilution 1 : 250) which binds to a region of human PrP downstream of the neurotoxic domain adjacent to helix region 2: residues 142–160 [25] (kindly provided by Prof. G. Hunsmann, The German Primate Centre, Gottingen, Germany). Immunoreactivity was enhanced by formic acid (98% for 5 minutes) and autoclaving (121°C for 10 minutes) pretreatment. Sections were treated with Dako Biotinylated Rabbit anti-Mouse (RAM) (dilution 1 : 100) and Dako ABComplex HRP kit for 45 minutes (Amersham, UK). Diaminobenzidine tetrahydrochloride was used as the chromogen. Immunostained sections were counterstained with haematoxylin for 1 minute to reveal neuronal cell bodies and glial cell nuclei.

### 2.3. Morphometric Methods

The distribution of the vacuolation, surviving neurons, glial cell nuclei, and PrP^sc^ deposits across the cortical lamainae was studied using methods similar to those of Duyckaerts et al. [26]. Five traverses from the pia mater to the edge of the white matter were located at random within each cortical area. With the exception of the synaptic PrP^sc^ deposits in sCJD, all pathological changes were counted in 50 × 250 *μ*m sample fields, the larger dimension of the field being located parallel with the surface of the pia mater. An eyepiece micrometer was used as the sample field and was moved down each traverse one step at a time from the pia mater to white matter. Histological features of the section were used to correctly position the field. Counts from the five traverses were added together to study the distribution of the pathology within each cortical region. Because of the diffuse nature of the synaptic deposits in sCJD, these lesions were quantified by “lattice sampling”, that is, by counting the number of times the intersections of the grid lines encountered a PrP^sc^ deposit [27]. 

### 2.4. Data Analysis

No attempt was made to locate precisely the boundaries between individual cortical laminae. First, the degree of pathological change and cell losses in the CJD cases made laminar identification difficult. Second, identification is especially difficult in the frontal cortex because it exhibits a heterotypical structure, that is, six laminae cannot be clearly identified even when the cortex is fully developed. Instead, variations in lesion density with distance below the pia mater were analysed using a curve-fitting procedure (STATISTICA software, Statsoft Inc., 2300 East 14th St, Tulsa, OK 74104, USA) [28, 29]. Hence, for each cortical region, a linear, quadratic, cubic, and quartic polynomial was fitted successively to the data. At each stage, the goodness of fit of the polynomial to the data was tested using correlation methods and analysis of variance. A more complex curve was accepted as a better fit to the data if it resulted in a significant increase in Pearson's correlation coefficient (“*r*”) and a significant reduction in the residual sums of squares compared with the preceding curves [28]. The distribution of the pathological changes in each cortical area was then classified according to whether a single (unimodal) or double (bimodal) peak of density was present (Table 2). If the distribution was unimodal, a further classification was made according to whether the density peak was located in the upper laminae, in the middle of the profile, or in the lower laminae. If a bimodal distribution was present, the data were further classified according to whether the upper density peak was greater than, equal to, or less than the lower density peak. There were several regions in which a pathological feature was distributed across the cortical laminae but also there were spared regions close to the pia mater and adjacent to the white matter. Differences in the frequency of the different patterns of distribution of each histological feature in the sCJD and vCJD cases were statistically tested using chi-square (*χ* ^2^) contingency table tests.

## 3. Results

**Figure 1:**
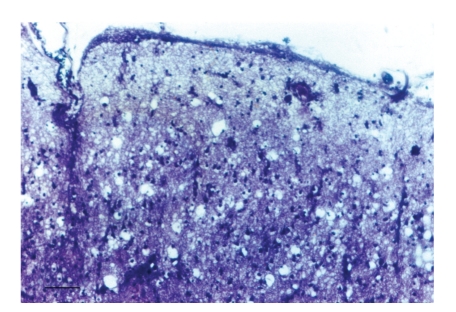
Vacuolation in the upper cortical laminae of the frontal cortex in a case of sporadic Creutzfeldt-Jakob disease (sCJD). (cresyl violet, magnification bar = 200 *μ*m).

**Figure 2:**
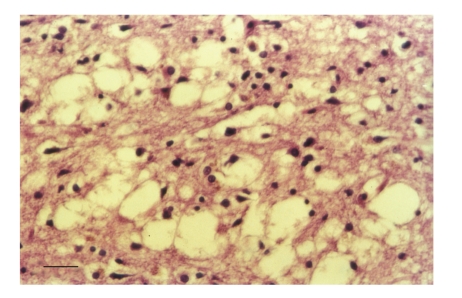
Vacuolation and gliosis in the occipital cortex in a case of sporadic Creutzfeldt-Jakob disease (sCJD), (H/E, magnification bar = 50 *μ*m).

**Figure 3:**
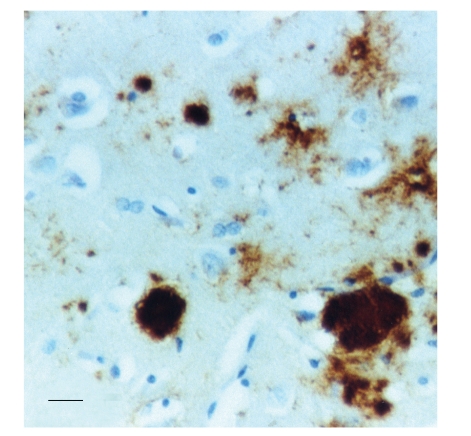
Prion protein (PrP^sc^) deposits in the frontal cortex of a case of variant Creutzfeldt-Jakob disease (vCJD), (PrP^sc^ immunohistochemistry, magnification bar = 50 *μ*m).

Figure 1 shows a low-power section of the frontal cortex of a case of sCJD showing the vacuolation affecting all laminae but especially the superficial cortical laminae. In Figure 2, a high-power section shows the vacuolation and gliosis in the lower laminae of the occipital cortex in a case of sCJD.  Figure 3 shows a typical section of the frontal cortex in a case of vCJD immunolabeled with antibodies raised against PrP^sc^ and reveals the strongly immunolabeled florid PrP^sc^ deposits which are composed of a condensed core of PrP^sc^ and the more lightly immunolabeled and irregularly shaped diffuse deposits.

**Figure 4:**
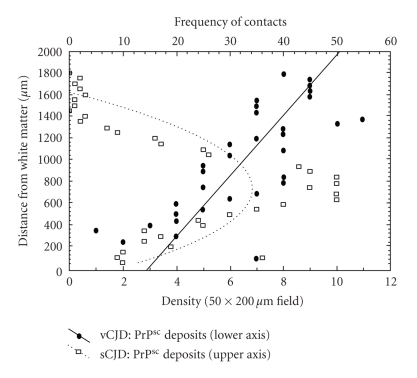
Distribution of the prion protein (PrP^sc^) deposits with distance across the cortical laminae in the cerebral cortex Creutzfeldt-Jakob disease (CJD). Diffuse deposits in variant CJD (vCJD), fit to polynomial (linear *r* = 0.82, *P* < .001); PrP^sc^ deposits in sporadic (CJD) (sCJD), fit to polynomial (second-order *r* = 0.81, *P* < .001).

Typical examples of the laminar distribution of the PrP^sc^ deposits in the CJD cases studied are shown in Figure 4. In vCJD (Case A, occipital cortex), the distribution of the diffuse PrP^sc^ deposits was fitted by a first-order polynomial (*r* = 0.82, *P* < .001) suggesting a linear decline in abundance with distance below the pia mater. By contrast, in sCJD (Case C, Parietal cortex), the distribution of the synaptic PrP^sc^ deposits was fitted by a second-order (quadratic) polynomial (*r* = 0.81, *P* < .001) suggesting a peak of abundance in the lower cortical laminae. 

A summary of the laminar distributions of the pathological changes in all brain areas is shown in Table 2. First, the distribution of the vacuolation in sCJD and vCJD was similar in many brain areas. Nevertheless, in sCJD there were a higher proportion of brain areas exhibiting vacuolation across all cortical laminae whereas in vCJD, there were a higher proportion of areas in which the vacuolation spared the superficial laminae and the region adjacent to the white matter. Second, in vCJD, the surviving neurons were more frequently evenly distributed across the cortex or exhibited a bimodal distribution in which the size of the upper and lower peaks was similar. By contrast in sCJD, in regions where a bimodal distribution of surviving neurons was present, the density peaks were asymmetric, the larger density peak occurring in the upper laminae. Third, the laminar distribution of the glial cell nuclei was similar in both sCJD and vCJD, the gliosis being most prominent in the lower cortex. Fourth, there were significant differences in the distribution of the PrP^sc^ deposits in sCJD and vCJD. The diffuse and florid PrP^sc^ deposits were more frequently distributed in the upper cortex in vCJD while the synaptic deposits were predominantly distributed in the lower cortex in sCJD. There were no essential differences in the laminar distribution of histological features in allocortical areas, for example, PHG and isocortical areas in either sCJD or vCJD.

## 4. Discussion

There were similarities and differences in the laminar distributions of the histological and pathological changes in sCJD and vCJD. First, there were similarities in the distribution of the vacuoles in the two disorders, but in sCJD, there were more brain regions in which the vacuoles were distributed across all laminae while in vCJD, there were more regions in which the vacuolation spared the superficial laminae and the region immediately adjacent to the white matter. Hence, the vacuolation may be more extensively distributed across the laminae in more regions in sCJD than in vCJD.

Second, there was a marked difference in the distribution of the surviving neurons in sCJD and vCJD. In normal control brain, cortical neurons are often bimodally distributed with peaks of density in the upper and lower cortex, the two peaks being asymmetric and the upper density peak being significantly larger than the lower density peak [18–20]. In sCJD, however, there were an increased number of brain regions in which the surviving neurons were present at a greater density in either the upper cortex alone or a bimodal distribution was present in which the density peak in the upper cortex was larger than in the lower cortex. In vCJD, surviving neurons were more frequently either uniformly distributed or in a bimodal distribution with equal-sized peaks. These results suggest greater neuronal loss in the upper cortex in vCJD and in the lower cortex in sCJD. 

Comparisons between sCJD and vCJD (*χ* ^2^ contingency tables): (a) All categories including totals for the bimodal distributions: Vacuoles *χ* ^2^ = 11.87 (5DF, *P* < .05); Neurons *χ* ^2^ = 13.27 (4DF, *P* < .05), Glial cells *χ* ^2^ = 4.43 (*P* > .05); Diffuse PrP^sc^ versus Synaptic PrP^sc^ *χ* ^2^ = 15.72 (4DF, *P* < .01), Florid PrP^sc^ versus Synaptic PrP^sc^ *χ* ^2^ = 17.94 (5DF, *P* < .001); (b) comparison of unimodal distributions only: Vacuoles *χ* ^2^ = 0.48 (2DF, *P* > .05); Neurons *χ* ^2^ = 3.12 (1DF, *P* > .05), Glial cells *χ* ^2^ = 3.23 (2DF, *P* > .05); Diffuse PrP^sc^ versus Synaptic PrP^sc^ *χ* ^2^ = 12.55 (1DF, *P* < .01), Florid PrP^sc^ versus Synaptic PrP^sc^ *χ* ^2^ = 13.37 (2DF, *P* < .01); (c) comparison of bimodal distributions only: Vacuoles *χ* ^2^ = 4.21 (2DF, *P* > .05), Neurons *χ* ^2^ = 7.56 (2DF, *P* < .05), Glia cell nuclei *χ* ^2^ = 0.67 (2DF, *P* > .05), Nonflorid PrP^sc^ *χ* ^2^ versus Synaptic PrP^sc^ *χ* ^2^ = 2.76 (2DF, *P* > .05), Florid PrP^sc^ versus Synaptic PrP^sc^ *χ* ^2^ = 2.76 (2DF, *P* > .05).

Third, there are similarities in the distribution of glial cell nuclei in sCJD and vCJD which showed a marked preference for the lower cortical laminae, a distribution also reported in previous studies [30]. Although this distribution could reflect greater pathological change in the lower cortex in sCJD, where it is accompanied by greater neuronal loss and PrP^sc^ deposition, this is unlikely to be the case in vCJD where neuronal losses appear to be greater in the upper cortical laminae. It is possible that in sCJD, the glial cell reaction directly reflects pathological changes in the lower laminae but in vCJD reflects greater degeneration of the afferent and efferent subcortical pathways which have their cells of origin or axon terminals in the lower cortex.

Fourth, significant differences in the laminar distribution of PrP^sc^ deposits were observed in the two disorders. In vCJD, the diffuse and florid PrP^sc^ deposits showed a marked preference for the upper cortical laminae while the synaptic deposits of sCJD were more frequently distributed in the lower cortex. These distributions could be related to the degeneration of different anatomical pathways in the neocortex. Hence, the distribution of the pathological changes in vCJD suggests degeneration of the feed-forward corticocortical projections while degeneration of the feed-back corticocortical pathways and/or the feedback pathways may be present in sCJD [21, 31]. 

Differences in the distribution of the pathology in sCJD and vCJD could reflect the differences in the aetiology, and subsequent spread of prion pathology. Hence in vCJD, prions are believed to enter the nervous system by absorption through the gut following consumption of infected food, replication in the spleen, and neural entry via the spinal cord [14] whereas sporadic cases are less likely to have an iatrogenic aetiology, and to result from either random mutation or posttranslational modification of the *PrP* gene [3, 4]. The different origins of the pathogenic prions may affect the subsequent development and spread of the pathology through the brain. In vCJD, for example, the pathology may spread into the cerebellum from the spinal cord via the anterior spinocerebellar tract [32]. Subsequent spread may then involve the loop of connections involving the thalamus, neocortex (especially the feed-forward connections), pons, and cerebellum [33]. By contrast, in sCJD, the pathological prions may be formed *in situ,* and the distribution of the pathology will reflect the site of origin and the pathways by which prion pathology spreads from these sites. Hence, if there are multiple sites of origin of the pathology, there may be a less marked topographic pattern to the pathology in sCJD than in vCJD.

## 5. Conclusions

The data are consistent with the hypothesis that there are different patterns of cortical degeneration in sCJD and vCJD. All laminae of the neocortex may be affected in both sCJD and vCJD, but, there is a greater development of the pathology in the lower laminae in sCJD and in the upper laminae in vCJD. Differences in cortical degeneration may be directly related to the different aetiologies of the two disorders and differences in the pattern of spread of the pathology through the brain. 

## Figures and Tables

**Table 1:** Demographic data and gross pathological features of the sporadic (sCJD) and variant Creutzfeldt-Jakob disease (vCJD) cases studied.

Case	Sex	Age at onset	Duration	Brain weight	Gross atrophy
		(years)	(years)	(gm)	
sCJD					
A	F	71	0.25	1222	Bilateral, diffuse
B	F	59	2.4	986	Severe Fr, T, C
C	F	71	0.16	1275	Mild Fr
D	F	71	0.25	1169	Mild
E	M	78	0.25	1562	Moderate
F	M	50	0.25	1292	None
G	M	67	0.16	1425	Moderate
H	F	69	0.16	1365	Mild
I	M	60	NA	1621	None
J	M	61	1	1270	Mild
K	F	78	0.40	1061	Mild, diffuse

vCJD					
A	F	39	2	586 L	None
B	F	28	1	1375	None
C	F	28	1	NA	NA
D	M	19	1	NA	NA
E	M	30	1	699 R	None
F	M	48	2	1470	None
G	F	34	1	810 L	None
H	M	18	1	1434	None
I	M	24	1	NA	NA
J	F	21	2	1394	None
K	M	35	1	718 R	None

Abbreviations: M: Male, F: Female, Fr: Frontal cortex, T: temporal cortex, C: cerebellum, R: Right hemisphere, L: Left hemisphere, NA = data not available.

**Table 2:** Frequency of particular types of distribution of the vacuolation, surviving neurons, glial cell nuclei and prion protein (PrP^sc^) deposition across the cortical laminae in the cerebral cortex of cases of sporadic Creutzfeldt-Jakob disease (sCJD) and variant CJD (vCJD).

Type of distribution										

					Unimodal			Bimodal		
Feature	Case	*N*	NS	RD	*U*	*M*	*L*	*U* > *L*	*U* = *L*	*U* < *L*
Vacuoles	sCJD	43	6	10	3	0	4	7	8	5
	vCJD	58	0	17	9	1	9	7	14	1
Neurons	sCJD	43	4	5	19	0	0	12	3	0
	vCJD	52	12	11	8	0	3	6	10	2
Glia cells	sCJD	42	1	1	0	0	37	0	1	2
	vCJD	57	3	3	2	2	44	0	2	1
PrP^sc^	sCJD	31	4	3	2	0	13	4	2	3
Diffuse PrP^sc^	vCJD	55	10	3	21	0	7	6	6	1
Florid PrP^sc^	vCJD	51	14	1	16	1	6	6	6	1

N: number of neocortical regions studied, NS: no significant difference in density with laminar depth, RD: restrictive distribution, sparing the superficial laminae and the region adjacent to white matter, U: upper cortical laminae, M: middle cortical laminae, L: lower laminae.
